# Chemotherapy Enriches for Proinflammatory Macrophage Phenotypes that Support Cancer Stem-Like Cells and Disease Progression in Ovarian Cancer

**DOI:** 10.1158/2767-9764.CRC-24-0311

**Published:** 2024-10-09

**Authors:** Luisjesus S. Cruz, Mikella Robinson, Denay Stevenson, Isabella C. Amador, Gregory J. Jordan, Sofia Valencia, Carolina Navarrete, Carrie D. House

**Affiliations:** 1 Department of Biology, San Diego State University, San Diego, California.; 2 Moores Cancer Center, University of California San Diego, San Diego, California.

## Abstract

High-grade serous ovarian cancer remains a poorly understood disease with a high mortality rate. Although most patients respond to cytotoxic therapies, a majority will experience recurrence. This may be due to a minority of drug-resistant cancer stem-like cells (CSC) that survive chemotherapy and are capable of repopulating heterogeneous tumors. It remains unclear how CSCs are supported in the tumor microenvironment (TME) particularly during chemotherapy exposure. Tumor-associated macrophages (TAM) make up half of the immune population of the ovarian TME and are known to support CSCs and contribute to cancer progression. TAMs are plastic cells that alter their phenotype in response to environmental stimuli and thus may influence CSC maintenance during chemotherapy. Given the plasticity of TAMs, we studied the effects of carboplatin on macrophage phenotypes using both THP1- and peripheral blood mononuclear cell (PBMC)–derived macrophages and whether this supports CSCs and ovarian cancer progression following treatment. We found that carboplatin exposure induces an M1-like proinflammatory phenotype that promotes *SOX2* expression, spheroid formation, and CD117^+^ ovarian CSCs, and that macrophage-secreted CCL2/MCP-1 is at least partially responsible for this effect. Depletion of TAMs during carboplatin exposure results in fewer CSCs and prolonged survival in a xenograft model of ovarian cancer. This study supports a role for platinum-based chemotherapies in promoting a transient proinflammatory M1-like TAM that enriches for CSCs during treatment. Improving our understanding of TME responses to cytotoxic drugs and identifying novel mechanisms of CSC maintenance will enable the development of better therapeutic strategies for high-grade serous ovarian cancer.

**Significance:**

We show that chemotherapy enhances proinflammatory macrophage phenotypes that correlate with ovarian cancer progression. Given that macrophages are the most prominent immune cell within these tumors, this work provides the foundation for future translational studies targeting specific macrophage populations during chemotherapy, a promising approach to prevent relapse in ovarian cancer.

## Introduction

Ovarian cancer is the most lethal gynecologic malignancy in the United States and the second most lethal gynecologic malignancy worldwide (1, 2). Nonspecific early symptoms and ineffective screening methods are largely responsible for the late-stage diagnosis occurring in more than 70% of ovarian cancer cases (3). Following diagnosis, debulking surgery and a combination of platinum- and taxane-based chemotherapies are the current standard of care. Although most patients show no evidence of disease after initial chemotherapy treatment, a majority of these patients relapse and develop chemotherapy-resistant disease within 2 years (4). Furthermore, more than half of patients diagnosed with advanced stage disease will die within 5 years following diagnosis (2). Mechanisms of acquired resistance following chemotherapy in recurrent ovarian cancer remain unclear and must be clarified to improve morbidity and mortality.

Studies suggest a minority subset of tumor cells, termed cancer stem-like cells (CSC), can evade cytotoxic chemotherapy and facilitate cancer recurrence (5–8). It is possible that the chemotherapy-resistant CSCs are pre-existing or, alternatively, cells with this phenotype may develop during disease progression. Ovarian cancer is a heterogeneous peritoneal disease, characterized by a dynamic tumor microenvironment (TME) rich with stromal and immune cells, and an abundance of cytokines, chemokines, and other secretory factors (9). Among the various immune cell populations within ovarian tumors, macrophages are the most abundant, exhibiting diverse morphologic and physiologic functions (10). Macrophages are known to be the most plastic cells of the hematopoietic system (11–15), playing important roles in development, homeostasis, tissue repair, and immunity. Their plasticity enables either proinflammatory, anti-tumorigenic functions commonly observed in early-stage tumors, or anti-inflammatory, pro-tumorigenic functions, commonly observed in late-stage tumors.

Chemokines, cytokines, and other secreted factors within tumors can facilitate the recruitment of circulating monocytic cells into tumors to become tumor-associated macrophages (TAM; ref. 16). Generally, TAMs are tissue-resident or bone marrow–derived macrophages that participate in the formation of the TME to facilitate tumor growth, invasion, metastasis, and drug resistance (17, 18). TAMs in ovarian tumors are predominantly associated with the M2-polarized anti-inflammatory phenotype, whereas the proinflammatory, anti-tumorigenic M1-polarized phenotype is less frequently observed in late-stage disease (19). Due to their inherent plasticity, TAM phenotypes can change in response to the TME, and the polarization of infiltrating monocytes might be altered in a chemotherapy-treated tumor. Given that ovarian cancer is highly responsive to cytotoxic chemotherapy, we sought to determine if chemotherapy directly modifies TAM phenotypes to support the maintenance of CSCs and ovarian cancer progression. We show that carboplatin treatment of macrophages elicits an M1-like proinflammatory phenotype *in vitro* and *in vivo*, and suppression of TAMs during carboplatin treatment inhibits expansion of CSCs and prolongs survival in a xenograft model of ovarian cancer. These studies highlight the dynamic responses of TAMs to cytotoxic drugs and suggest new mechanisms of CSC maintenance and ovarian cancer relapse.

## Materials and Methods

### General cell culture conditions

CAOV4 (RRID: CVCL_0202) and OVCAR8 (RRID: CVCL_1629) cells were obtained and authenticated from NCI-Frederick DCTD tumor/cell line repository. OV90 (RRID: CVCL_3768) cells were obtained and authenticated by ATCC. THP1 (RRID: CVCL_0006) cells were obtained from Dr. Angelica Riestra at San Diego State University. Peripheral blood mononuclear cells (PBMC) were isolated from human buffy coat obtained from the San Diego Blood Bank. All cancer cells were maintained in RPMI (Gibco) supplemented with 10% FBS (Gibco) and 1% 10,000 U/mL penicillin/streptomycin (Gibco). THP1 cells were maintained in RPMI media supplemented with 10% heat inactive FBS (Gibco), 10 mmol/L HEPES (Gibco), 1 mmol/L sodium pyruvate (Gibco), 4.5 g/L glucose stock (Gibco), and 0.05 mmol/L β-mercaptoethanol (Thermo Fisher Scientific). PB-macrophages were maintained in RPMI supplemented with 10% FBS and 2 mmol/L GlutaMAX (Gibco). Cells are tested for mycoplasma annually. Cells were maintained in culture for a maximum of 15 passages. For *in vitro* experiments with carboplatin treatment, all cells were exposed to 100 or 275 µmol/L carboplatin (Tocris Bioscience; Cat. # 2626) or vehicle in respective cell culture medium for 48 hours. For *in vitro* experiments with CCL2/MCP-1 treatment, all cells were exposed to 10 ng/mL (R&D; Cat. # 279-MC) or vehicle in respective cell culture medium for 48 hours.

### Isolation of peripheral blood monocyte and differentiation to macrophage

Adult human blood was obtained from anonymous female donors through the San Diego Blood Bank. PBMCs were isolated by Ficoll-Paque Plus (GE Healthcare) density-gradient centrifugation from heparinized buffy coats. Monocytes (PB-monocyte) were then isolated by CD14 positive selection using CD14 MicroBeads (Miltenyi Biotech; Cat. # 130-020-201) or straight from Buffy Coat CD14 MicroBead Kit (Miltenyi Biotech; Cat. # 130-114-976) according to manufacturer’s instructions and then further differentiated to macrophages (PB-macrophages) in PB-macrophage media supplemented with 100 ng/mL macrophage colony-stimulating factor (M-CSF, PeproTech; Cat. # 300-25) for 7 days.

### Macrophage polarization

Human THP1 cells were differentiated to M0 macrophages for 48 hours in the presence of 100 nmol/L phorbol 12-myristate 13-acetate (PMA, Sigma-Aldrich; Cat. # P1585) whereas PB-monocytes were differentiated to M0 macrophage for 7 days in the presence of 100 ng/mL M-CSF (PeproTech; Cat. # 300-25). M0 macrophages were then polarized to either M1 or M2 macrophages for 48 hours with different stimuli: 50 ng/mL LPS for THP1 and 100 ng/mL LPS for PB-derived and 20 ng/mL IFNγ (PeproTech; Cat. # 300-02) for M1 polarization or 20 ng/mL IL4 (PeproTech; Cat. # 200-04) and 20 ng/mL IL13 (PeproTech; Cat. # 200-13) for M2 polarization. The effect of activation was evaluated by quantifying changes in different phenotypic markers by flow cytometry for M0: hCD68-APC-cy7 (1:16, BioLegend; Cat. # 333822, RRID: AB_2571965), for M1: hCD80-APC (1:20, BioLegend; Cat. # 305220, RRID: AB_2076147), and for M2: hCD206-PE (1:20, BioLegend; Cat. # 321106, RRID: AB_571911); qRT-PCR; and ELISA.

### Macrophage viability, pyroptosis, and apoptosis

Macrophages were plated at approximately 40,000 cells/well on 96-well plates and differentiated to M0 and then polarized to M1 and M2 populations described above. Macrophages were then treated with or without carboplatin (275 µmol/L) for 48 hours and analyzed for viability through ATP activity using CellTiter-Glo 2.0 Cell Viability Assay (Promega; Cat. # G9241) following manufacturer’s protocol. Pyroptosis activity was assessed through caspase-1 activity using Caspase-Glo 1 Inflammasome Assay (Promega; Cat. # G9951) following manufacturer’s protocol. Apoptosis was evaluated through caspase-3/7 activity using Caspase-Glo 3/7 Assay (Promega; Cat. # G8091) following manufacturer’s instructions. All samples were analyzed for luciferase activity using Thermo Fisher Scientific Varioskan.

### Co-culture experiments

Transwell inserts with a 0.4 μm porous membrane (Thermo Fisher Scientific; Cat. # 141078) were used for indirect co-culture of THP1- or PB-derived macrophages with CAOV4 cells. Macrophages were seeded at 750,000 cells/mL into the bottom well of a transwell plate and subsequently differentiated and polarized into M1 and M2 phenotypes as described above. Simultaneously, CAOV4 or OVCAR8 cells were seeded at 150,000 cells/mL into transwell inserts and incubated overnight to adhere to insert membranes. Following polarization, transwell inserts containing adherent CAOV4 or OVCAR8 cells were carefully transferred to transwell plates containing either M0, M1, or M2 macrophages yielding an ovarian cancer:macrophage co-culture ratio of 1:5. Cocultures were allowed to rest for 6 hours prior to treatments. After 48 hours, macrophages were collected for flow cytometry as described below for M1: hCD80-APC (1:20, BioLegend; Cat. # 305220, RRID: AB_2076147), and for M2: hCD206-PE (1:20, BioLegend; Cat. # 321106, RRID: AB_571911), and ovarian cancer cells were collected for RNA as described below. Conditioned media were collected from all samples and stored in ˗80°C for downstream experiments.

### Spheroid formation assay

Cells were plated at 500 cells/well in ultra-low attachment, flat bottom 96-well plates using media as described in the figure legends. Spheroids were allowed to grow for 5 to 6 days, followed by staining with Hoechst 33342. Spheroid size 30 to 500 μm were counted using ImageXpress Pico and CellReporterXpress software. Spheroid formation is defined as (# of spheroids)/(# of cells per well).

### *In vivo* studies

All animal studies were approved by the SDSU Animal Care and Use Committee (protocol approval number 21-05-003H). For subcutaneous xenografts, 500,000 OV90 cells in 1:1 Matrigel in PBS were subcutaneously injected into the left flank of 8-week-old female immunodeficient nude mice (NU/J, The Jackson Laboratory; RRID: IMSR_JAX:002019). Mice weight and tumor measurements were performed in a blind fashion twice weekly. Once tumors reached a size of 150 mm^3^, mice were treated with intraperitoneal (i.p.) injections of either vehicle or carboplatin (50 mg/kg) once per week for 3 weeks. Mice were sacrificed either (i) on average 5 days after the third dose of carboplatin or (ii) at full growth of tumors reaching a volume of 3000 mm^3^ to evaluate progressive changes.

For macrophage depletion studies, we used 6-week-old female immunodeficient nude mice (NU/J, The Jackson Laboratory; RRID: IMSR_JAX:002019) and intraperitoneal injected CAOV4 human ovarian cancer cells at a concentration of 2 million cells per mice. Tumors were allowed to develop for 4 days before starting treatment with either: (i) vehicle + BLZ945 vehicle (by oral gavage 5 days per week), (ii) carboplatin (50 mg/kg, four doses, 1 day per week) + BLZ945 vehicle (by oral gavage 5 days per week), or (iii) carboplatin (50 mg/kg, four doses, 1 day per week) + BLZ945 inhibitor (200 mg/kg oral gavage 5 days per week). To ensure macrophage depletion, BLZ945 inhibitor (MedChemExpress; Cat. # HY-12768) or BLZ945 vehicle was administered 24 hours before chemotherapy and again every day following chemotherapy for 5 days. Mice were monitored every week for clinical signs of disease progression. The residual tumor group was collected 3 days following last carboplatin treatment, and the regrown tumor group was collected 120 days following the last carboplatin treatment or at humane endpoints. Macrophage depletion was verified from single-cell dissociation of excised omental tumors and analyzed via flow cytometry. Mice tumor tissues were also collected and analyzed for secretome using LEGENDplex and for CSC expression by flow cytometry or Sox2 expression by IHC as described below.

### Flow cytometry

*In vivo* omental tumor tissue was excised and mechanically dissociated to prepare for flow cytometry analysis. Briefly, tumor samples were cut into small pieces of 2 to 4 mm then transferred into a gentleMACS C Tube containing the enzyme mix attached to gentleMACS Dissociator using a Human Tumor Dissociation Kit (Miltenyi Biotech; Cat. # 130-095-929) as per manufacturer’s protocol. After dissociation, large particles were removed by 70 μm mesh strainer, and cells were treated with ammonium–chloride–potassium (ACK) lysing buffer to lyse red blood cells. Cells were then treated with Fc receptor blocker (Miltenyi Biotech; Cat. # 130-059-901) for 10 minutes in 2°C to 8°C followed by flow cytometry antibody staining for two subsequent panels TAM-M1 and TAM-M2: for total TAMs, the antibodies included mCD45-PerCP-Cy5.5 (1:160, BioLegend; Cat. # 103132, RRID: AB_893340), mCD11b-PE/Dazzle594 (1:666, BioLegend; Cat. # 101256, RRID: AB_2563648), and mF4/80-APC-Cy7 (1:20, BioLegend; Cat. # 123118, RRID: AB_893477); and for M1, the antibodies included mMHC-II-PE (1:160, BioLegend; Cat. # 107608, RRID: AB_313323), mCD80-APC (1:40, BioLegend; Cat. # 104714, RRID: AB_313135), and mCD86-AlexaFluor488 (1:50, BioLegend; Cat. # 105018, RRID: AB_493462) and for M2, the antibodies included mCD206-AlexaFluor488 (1:100, BioLegend; Cat. # 141710, RRID: AB_10900445), mCD273-APC (1:40, BioLegend; Cat. # 107210, RRID: AB_2566345), and mCD163-PE (1:80, BioLegend; Cat. # 155308, RRID: AB_2814062). Samples were stained for 20 minutes and incubated at 4°C prior to fixing following manufacturer’s protocol (Cyto-Fast Fix/Perm Buffer Set, BioLegend; Cat. # 426803). Flow cytometry analysis of CSCs was performed using the same dissociated tumor samples. Briefly, cells were stained for human CD117 (1:50, Miltenyi Biotec; Cat. # 130-111-593, RRID: AB_2654579) and human CD133 (1:50, Miltenyi Biotec; Cat. # 130-110-962, RRID: AB_2654888), and ALDH activity was analyzed via ALDEFLUOR Kit following manufacturer’s instructions (STEMCELL Technologies; Cat. # 01700).

Cells were collected from *in vitro* samples and treated with Fc receptor blocker (Miltenyi Biotec; Cat# 130-059-901, RRID: AB_2892112) for 10 minutes in 2°C to 8°C followed by antibody staining for M1: hCD80-APC (1:20, BioLegend; Cat. # 305220, RRID: AB_2076147), for M2: hCD206-AlexaFluor488 (1:20, BioLegend; Cat. # 321114, RRID: AB_571875), and for viability SYTOX Blue (Thermo Fisher Scientific; Cat. # S34857). Samples were fixed and permeabilized using Cyto-Fast Fix/Perm Buffer Set following manufacturer’s protocol (BioLegend; Cat. # 426803) prior to analysis. All flow cytometry was performed using BD FACSMelody and analyzed via FlowJo 10.9.0 or an Amnis ImageStream MkII and analyzed via IDEAS 6.2.

### RNA extraction and qRT-PCR

Total RNA was isolated using the NucleoSpin RNA Plus kit (MACHEREY-NAGEL, Inc.; Cat. # 740984) or Direct-zol RNA Miniprep Plus Kits (ZYMO RESEARCH; Cat. # R2072) or E.Z.N.A. MicroElute Total RNA Kit (Omega Bio-tek; Cat. # R6831) as per the manufacturer’s instructions. cDNA synthesis was carried out using the High-Capacity cDNA Reverse Transcription kit (Applied Biosystems; Cat. # 4368814) as per the manufacturer’s protocol. Quantification and normalization of gene expression were performed using TaqMan Fast Advanced Master Mix and TaqMan probes. Gene probes were purchased from Thermo Fisher Scientific (*TNFα*, Cat. # Hs00174128_m1; *IL1β*, Cat. # Hs01555410_m1; *IL10*, Cat. # Hs00961622_m1; *TGFβ1*, Cat. # Hs00998133_m1; *CCL22*, Cat. # Hs01574247_m1; *SOX2*, Cat. # Hs01053049_s1; *OCT4*, Cat. # Hs04260367_gH; *NANOG*, Cat. # Hs02387400_g1; *GAPDH*, Cat. # Hs02786624_g1). Experiments were run on a QuantStudio 3 instrument and analyzed with the QuantStudio Design and Analysis software using the delta-delta Ct method with GAPDH endogenous control.

### ELISA and LEGENDplex

Macrophage secreted factors were analyzed by ELISA for TNFα (R&D; Cat. # DY210-05), IL1β (R&D; Cat. # DY201-05), IL10 (R&D; Cat. # DY217B-05), and TGFβ1 (R&D; Cat. # DY240-05) using supernatant from M0, M1, and M2 macrophage according manufacturer’s instructions. Supernatants were also used to evaluate an extended panel of secreted factors via multiplex assays for human essential immune response (BioLegend; Cat. # 740929) and human TNFSF family proteins (BioLegend; Cat. # 741309) following manufacturer’s protocol. Tumor samples resected from mice were homogenized and proteins collected for mouse inflammation panel (BioLegend; Cat. # 740446) following manufacturer’s protocol. Briefly, tumor tissues were weighed, and appropriate volume of NP-40 Lysis Buffer (supplemented with HALT Protease and Phosphatase inhibitor cocktail) was added, followed by dissociation using gentleMACS M Tube on gentleMACS dissociator as directed by the manufacturer. Supernatants were collected after centrifugation and evaluated for secreted factors on the LEGENDplex Mouse Immune Response panel (BioLegend; Cat. # 740150).

### Immunohistochemistry (IHC)

Tumors were resected, fixed in 10% neutral buffered formalin, and stored in 70% ethanol before processing. Tumors were embedded in paraffin and sectioned at 5 μm. Antigen retrieval was performed in citrate buffer and quenched with hydrogen peroxide. Slides were incubated with a primary antibody for Sox2 (1:300, Cell Signaling Technology; Cat. # 3579, RRID: AB_2195767) overnight at 4°C followed by a horseradish peroxidase (HRP)–linked secondary (Cell Signaling Technology; Cat. # 8114, RRID: AB_10544930) for 1 hour at room temperature and processed using 3,3′-diaminobenzidine (DAB) kit from Vector Laboratories. Four randomly selected images per slide were acquired with a ZEISS Primo Star HAL/LED Microscope and imaged using ToupView. A digital quantification of DAB staining was performed using ImageJ with a FIJI deconvolution package as described previously (20).

### Statistical analysis

Statistics were generated using Prism 10.0.3 with data acquired from at least three independent biological replicates. Results are presented as mean ± SEM. Significance was calculated using either unpaired *t* test for two means or either a one-way ANOVA or two-way ANOVA for comparisons of three or more means with a *post hoc* test to identify differences between groups as described in figure legends. Differences between means are considered statistically significant at the 95% level (*P* < 0.05). Dose response was assessed using a least squares nonlinear regression to calculate the curve and IC_50_ values. Statistics were completed as described here or as otherwise noted in the figure legends.

### Data availability

The data generated in this study are available within the article and its Supplementary Materials. Raw data files are available upon request from the corresponding author.

## Results

Given the pivotal role that macrophages play in supporting ovarian cancer progression, we sought to characterize the phenotypic changes of macrophages in response to cytotoxic treatment with carboplatin and distinguish how these changes influence CSC maintenance. We first differentiated THP1- and primary PBMC-derived monocytes to M0 macrophage and subsequently polarized to M1-like or M2-like macrophage phenotypes (Supplementary Fig. S1A and S1B) and compared all populations. Quantification of the M1 marker CD80 and the M2 marker CD206 indicated that all populations express both markers; however, the M1-like macrophages express a significantly higher level of CD80, whereas M2-like macrophages express a higher level of CD206 (Supplementary Fig. S1A and S1B). Interestingly, the M0 population had an expression pattern similar to M2-like cells in THP1 cells. The shared expression of these M1 and M2 markers following polarization is observed in many studies (16, 21) and may reflect the spectrum of phenotypes associated with macrophage subpopulations. To further distinguish the respective phenotypes resulting from our polarization protocol, we assessed gene expression and secreted proteins commonly associated with M1-like or M2-like macrophages. As expected, expression and/or production of TNFα and IL1β was highest in the M1-like population from both THP1- and PBMC-derived macrophages (Supplementary Fig. S1C and S1D). Similarly, expression and/or production of TGFβ1 and CCL22 was highest in the M2-like population (Supplementary Fig. S1C and S1D). IL10 production, a phenotype associated with M1 or M2 macrophages (22, 23), was detected in both M1-like and M2-like THP1-derived macrophages, but primarily from M1-like PBMC-derived macrophages. These data indicate that our differentiation and polarization procedures enrich for the phenotypes we are seeking to investigate; henceforth, we will refer to the M1-like polarization as “M1” and the M2-like polarization as “M2” as a simplification of macrophage subtypes within a spectrum of phenotypes (24, 25).

**Figure 1:**
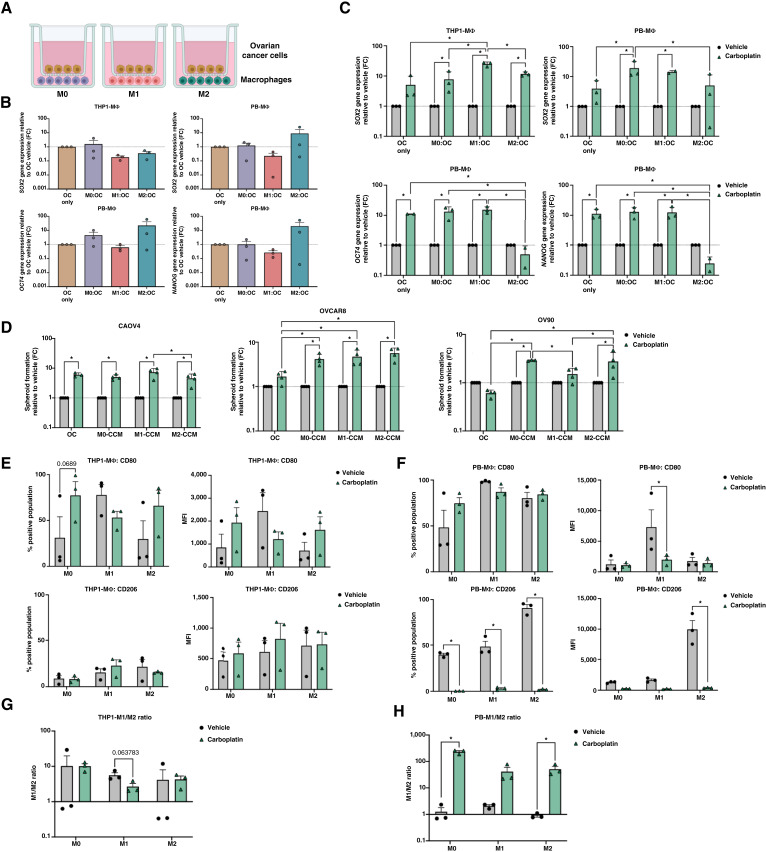
Macrophages enhance SOX2 expression in CAOV4 cells during chemotherapy exposure. **A,** Schematic of indirect co-culture experiment with THP1- or PBMC-derived macrophages and CAOV4 cells. Briefly, macrophages (MΦ) and CAOV4 cells (OC) were plated on bottom wells and top wells, respectively, and subsequently treated with carboplatin (275 µmol/L) for 48 hours. **B** and **C,** qPCR analysis of *SOX2*, *OCT4*, and *NANOG* gene expression in CAOV4 cells co-cultured with macrophages **(B) **compared with cancer cells alone one-way ANOVA and Tukey *post hoc* test, or **(C)** in the presence of carboplatin (275 µmol/L) relative to same condition vehicle two-way ANOVA and Tukey *post hoc* test. **D,** Spheroid formation ability of ovarian cancer cells after 48 hours of treatment with CCM relative to same condition vehicle. Two-way ANOVA, Tukey* posthoc* test. **E** and **F,** Flow cytometry analysis of THP1- (**E**) and PBMC-derived (**F**) macrophages for expression of M1-CD80 and M2-CD206 after 48 hours of carboplatin treatment (275 µmol/L) compared with vehicle in co-culture with CAOV4 cells. Unpaired *t* test. Mean and SD. **G** and **H,** M1/M2 ratio of THP1- (**G**) and PBMC-derived (**H**) macrophages calculated from **E** and **F**. Unpaired *t* test. Mean and SEM. *, *P* < 0.05, no stars indicate ns. MFI, mean fluorescence intensity.

We have previously shown that *SOX2* is a marker of chemoresistance and recurrence in ovarian cancer (26); thus, we next sought to characterize changes in *SOX2*, *OCT4*, and *NANOG* expression in ovarian cancer cells indirectly co-cultured with differentially polarized macrophage populations (Fig. 1A). Briefly, we first seeded THP1- or PBMC-derived macrophages (MΦ) in the bottom well of a transwell plate and then polarized to M1 or M2 phenotypes. We subsequently seeded CAOV4 or OVCAR8 ovarian cancer (OC) cells on the top well and allowed for acclimation of the co-culture for 6 hours before carboplatin treatment. We found that exposure to M0, M1, or M2 macrophages for 48 hours did not significantly affect *SOX2*, *OCT4*, and *NANOG* expression in CAOV4 (Fig. 1B) or OVCAR8 ovarian cancer cells (Supplementary Fig. S2A). However, treatment with an IC_50_ dose of carboplatin led to enhanced expression of *SOX2*, *OCT4*, and *NANOG* in cancer cells seeded alone, in agreement with our previous work (Fig. 1C; Supplementary Fig. S2B; ref. 26). *SOX2* expression was significantly increased in the presence of M0, M1, or M2-like macrophages and carboplatin compared with their same condition vehicle and in presence of M0 (PBMC-derived) or M1 (THP1) compared with cancer cells alone with carboplatin (Fig. 1C; Supplementary Fig. S2B). We also found that cancer cells treated with conditioned media from the cocultures (CCM) had no consistent enhancement of spheroid formation using vehicle CCM (Supplementary Fig. S2C), but had significantly enhanced spheroid formation using carboplatin CCM compared with same treatment vehicle and in some cases compared with cancer cells alone with carboplatin (Fig. 1D).

We next assessed changes in the M1 (CD80) or M2 (CD206) markers in M0, M1-like, or M2-like macrophages collected from the co-culture conditions. Exposure to cancer cells for 48 hours in vehicle conditions maintained THP1-derived macrophages with traditional marker expression, with 30% M0 cells expressing CD80 and 10% expressing CD206, 60% M1 cells expressing CD80, and 26% M2 cells expressing CD206 (Fig. 1E; Supplementary Fig. S2D). Relative to monoculture (Supplementary Fig. S1B), a majority of the PBMC-derived macrophages displayed CD80 marker upon co-culture with ovarian cancer cells (Fig. 1F; Supplementary Fig. S2E). Carboplatin exposure led to an increase or maintenance of CD80 expression in M0 and M2 THP1-derived macrophage populations and a decrease in CD206 expression in all populations derived from PBMC-derived macrophages (Fig. 1E and F; Supplementary Fig. S2D and S2E). Both scenarios ultimately led to a higher or consistent M1/M2 ratio in either cell type (Fig. 1G and H). These data suggest that chemotherapy potentially alters macrophage phenotypes and enhances expression of stemness pathways in ovarian cancer cells.

**Figure 2:**
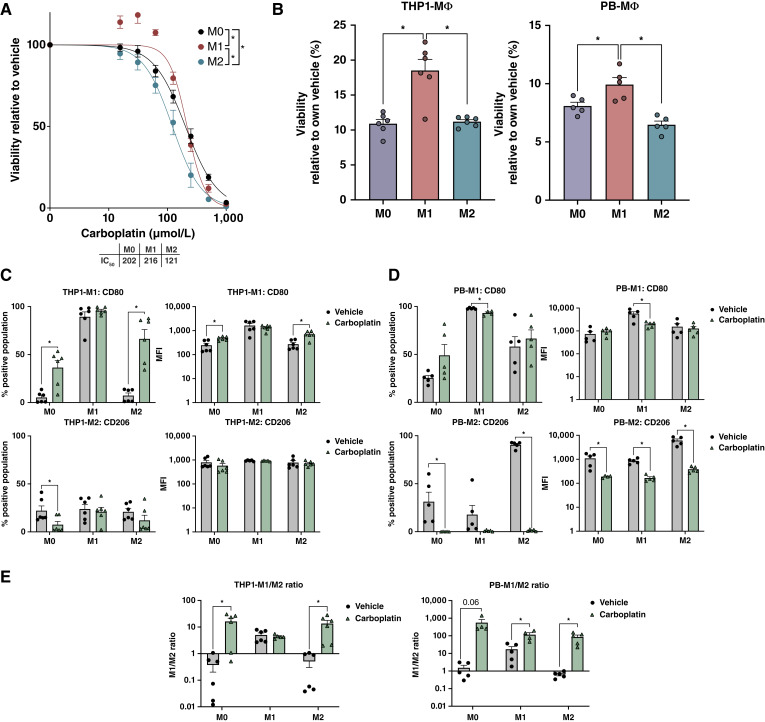
M1 macrophages are more resistant to carboplatin. **A,** Dose curves for THP1-derived macrophages after 48 hours of carboplatin treatment. Two-way ANOVA and Tukey *post hoc* test comparing main effects between M0, M1, and M2, *n* = 3. **B,** Viability of THP1- and PBMC-derived macrophages after 48 hours of carboplatin treatment (275 µmol/L) compared with vehicle. One-way ANOVA and Tukey *post hoc* test. **C** and **D,** Quantification of M1-CD80 and M2-CD206 expression by flow cytometry in THP1- (**C**) and PBMC-derived macrophages (**D**) after 48 hours of carboplatin treatment (275 µmol/L). Unpaired *t* test. Mean and SD. **E,** M1/M2 ratio after 48 hours of carboplatin treatment (275 µmol/L) compared with vehicle. Unpaired *t* test. Mean and SEM. *, *P* < 0.05, no stars indicate ns. MFI, mean fluorescence intensity.

To better understand the effect of cytotoxic chemotherapy on macrophage phenotypes, we calculated the carboplatin IC_50_ values of the different macrophage populations and discovered that M2 macrophages were most sensitive to carboplatin relative to M1 macrophages (Fig. 2A). We proceeded by treating the different macrophage populations with carboplatin concentration corresponding to the CAOV4 ovarian cancer cell IC_50_ for 48 hours in monoculture conditions and assessed viability and changes in M1 and/or M2 marker expression. Cancer cells have a carboplatin IC_50_ concentration of 275 µmol/L, which is far greater than that measured for the macrophages; therefore, we desired to understand how a higher carboplatin concentration potentially impacts macrophage phenotypes. As expected, CellTiter-Glo viability assays for both THP1- and PBMC-derived macrophages revealed that M1-polarized cells had the greatest resistance to carboplatin (Fig. 2B). Interestingly, THP1 populations treated with carboplatin maintained or increased expression of the M1 marker, CD80, relative to vehicle (Fig. 2C), whereas expression of the M2 marker, CD206, remained relatively unchanged or decreased (Fig. 2C). Conversely, M1 marker expression for PBMC-derived macrophage populations remains relatively unchanged (Fig. 2D) and M2 marker expression significantly decreases when treated with carboplatin (Fig. 2D). Similar to what we found in the co-culture system, upon 48 hours of carboplatin exposure, there was an overall increase in the M1/M2 ratio (Fig. 2E). We repeated this experiment at a physiologic carboplatin concentration of 100 µmol/L and saw a similar effect, with an overall increase in the M1/M2 ratio for M0 and M2 macrophages and a persistently high M1/M2 ratio for M1 macrophages (Supplementary Fig. S2F and S2G).

**Figure 3:**
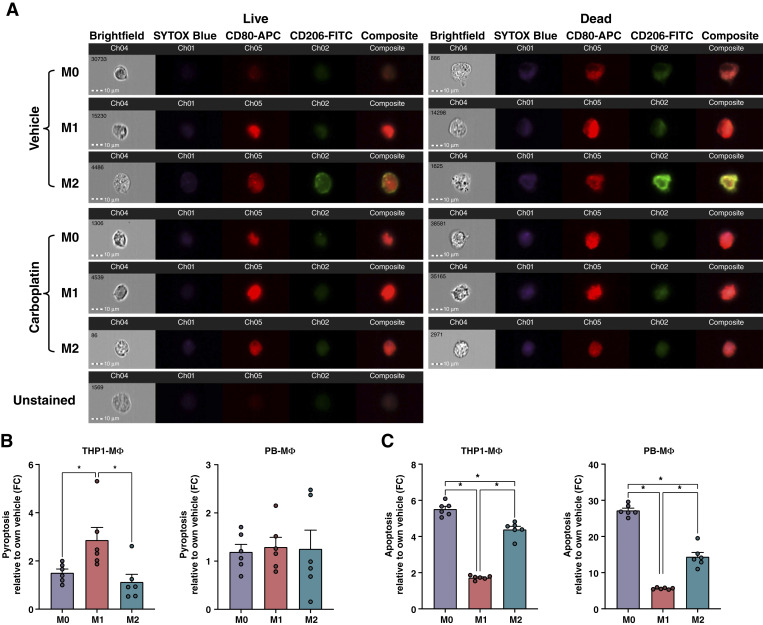
M1 macrophages are more resistant to chemotherapy-induced apoptosis. **A,** ImageStream analysis of PBMC-derived macrophages for brightfield, viability, M1-CD80, and M2-CD206 expression after 48 hours of carboplatin treatment (275 µmol/L). **B** and **C,** Caspase-1 activity to measure pyroptosis (**B**) and caspase-3/7 to measure apoptosis (**C**) in THP1- and PBMC-derived macrophages following 48 hours of carboplatin treatment (275 µmol/L) compared with vehicle. One-way ANOVA and Tukey *post hoc* test. Mean and SEM. *, *P* < 0.05, no stars indicate ns.

For a comprehensive visualization of macrophage phenotypes following carboplatin exposure, we performed imaging flow cytometry of PBMC-derived macrophages and quantified both viability and, M1 and M2 cell surface stains (Fig. 3A). We found that live cells, identified by SYTOX Blue and brightfield imaging of cell structure integrity, confirmed expression of M1 and M2 markers seen by standard flow cytometry (Fig. 2D). SYTOX Blue staining and loss of cell structure integrity by brightfield suggest regulated cell death such as apoptosis or pyroptosis. We next investigated whether macrophages were undergoing pyroptosis or apoptosis in response to carboplatin using either a caspase-1 or a caspase- 3/7 activity assay, respectively. Pyroptosis is triggered by proinflammatory signals and, in contrast to apoptosis, often leads to cell rupture and spilling of intracellular contents. We found that THP1-derived M1 macrophages had significantly higher levels of caspase-1 than M0 or M2 macrophage, indicating increased pyroptosis relative to vehicle-treated cells (Fig. 3B). The PBMC-derived macrophage populations, however, had comparable levels of caspase-1 activity which were not significantly different from vehicle (Fig. 3B). Cleaved caspase-3/7 levels (Fig. 3C) were significantly lower in M1 macrophage populations from both cell types, indicating a higher resistance to carboplatin-induced apoptosis, in agreement with viability studies in Fig. 2B. Notably, the THP1 cells are overall more resistant to carboplatin, which may be due to their malignant properties relative to PBMC-derived macrophages from healthy donors.

**Figure 4:**
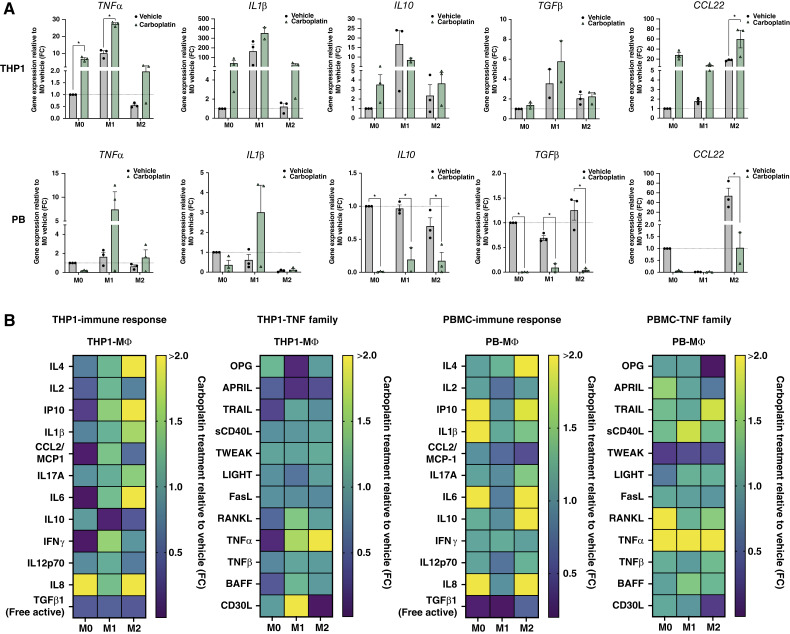
Carboplatin induces proinflammatory macrophage phenotypes. **A,** Gene expression *TNFα*, *IL1β*, *IL10*, *TGFβ*, and *CCL22* in THP1- and PBMC-derived macrophages following 48 hours of carboplatin treatment (275 µmol/L) relative to M0 vehicle. Unpaired *t* test. **B,** Protein expression of LEGENDplex Immune Response panel and TNF family panel of THP1- (left) and PBMC-derived macrophage (right) supernatants after 48 hours of carboplatin treatment (275 µmol/L) relative to vehicle. *n* = 3. Actual concentrations are in Supplementary Fig. S3 (see bar graphs). Mean and SEM. *, *P* < 0.05, no stars indicate ns.

To better assess phenotypic changes induced by carboplatin, we investigated alterations in factors secreted from the different macrophage populations in response to 48 hours of carboplatin exposure. We first confirmed gene expression changes associated with M1 or M2 that we showed in Supplementary Fig. S1 when validating polarization. *TNF*α and *IL1β* were consistently upregulated in M1 macrophages derived from both THP1 and PBMC cell types in response to carboplatin (Fig. 4A). Whereas there was little effect on *IL10* or *TGF*β in THP1 cells treated with carboplatin, these genes were significantly downregulated in PBMC-derived macrophages relative to vehicle (Fig. 4A). Finally, *CCL22* expression was upregulated in THP1-derived macrophages but downregulated in PBMC-derived macrophages treated with carboplatin relative to vehicle. Although THP1 cells showed a general increase in expression of all genes interrogated, the fold changes were highest for *IL1β*, an established M1 marker. Collectively, these data indicate an upregulation of M1 factors in carboplatin-treated macrophages relative to vehicle.

To gain a more comprehensive understanding of the phenotypic changes endured by macrophages treated with carboplatin, we used human immune response and TNF family cytokine protein panels to perform multiplex analyses of supernatants from vehicle- or carboplatin-treated macrophages. We first confirmed the baseline secretory profiles of macrophages immediately following differentiation and/or polarization (Supplementary Fig. S3). We then assessed changes in secreted factors after 48 hours of exposure to carboplatin relative to vehicle. Overall, the THP1-derived macrophages showed increased secretion of immune response cytokines in both the M1 and M2 macrophages. However, there was little change in the TNF family of cytokines, with the exception of decreased OPG and APRIL and increased CD30L in the M1 population, and increased TNFα in the M2 population (Fig. 4B; Supplementary Fig. S4A and S4B). The PBMC-derived macrophages had an increased secretion of both immune response and TNF family cytokines which were mostly observed in the M0 and M2 populations and involved in the upregulation of proinflammatory cytokines (Fig. 4B; Supplementary Fig. S4A and S4B). Interestingly, both THP1 and PBMC cell types displayed significant changes in secretion profiles primarily associated with the M0 or M2 macrophages, populations that we found were altered during chemotherapy (Fig. 2E). These data suggest that M1 macrophages may be relatively stable in their phenotype, and carboplatin chemotherapy elicits a proinflammatory response from M0 and/or M2 macrophages.

**Figure 5:**
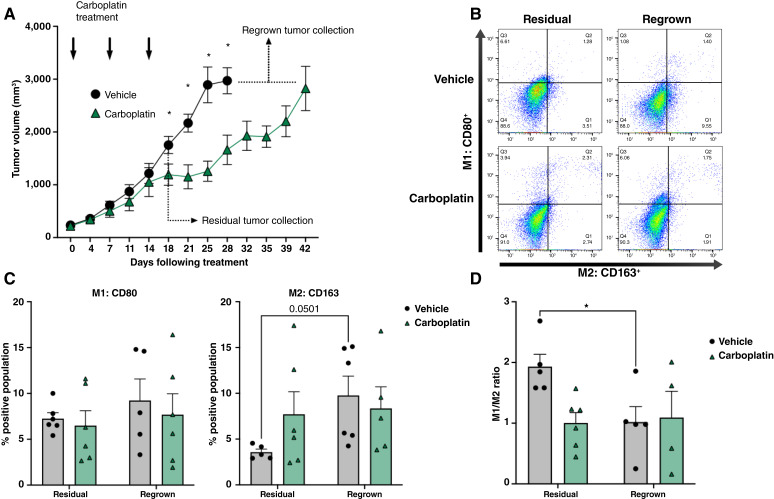
M1 macrophages persist following carboplatin regimen in subcutaneous models. **A,** Subcutaneous tumors were excised 4 days following three cycles of carboplatin or when maximum volume was reached. **B** and **D,** Dissociated tumors were analyzed by flow cytometry to determine M1-CD80 and M2-CD163 macrophage populations. **B,** Representative gates. **C,** Percent positive cells for M1-CD80 (left) or M2-CD163 (right) in vehicle or carboplatin-treated tumors. Two-way ANOVA and Sidak *post hoc* test. Mean and SD. **D,** M1/M2 ratio in tumors following carboplatin treatment of residual and regrown tumors. Ratio is calculated using positive population percent, as determined by flow cytometry. Two-way ANOVA and Sidak *post hoc* test. Mean and SEM. *, *P* < 0.05, no stars indicate ns.

To better appreciate the impact of carboplatin on macrophage phenotypes and their contribution to CSC maintenance, we investigated these phenotypes in human xenograft models of ovarian cancer. Using a subcutaneous mouse model, we analyzed changes in M1 (CD80) or M2 (CD163) markers in tumors resected either three days following three cycles of intraperitoneal carboplatin treatment, called "residual", or after tumors reformed following chemotherapy administration and reached endpoint criteria, called "regrown" (Fig. 5A and B). Interestingly, there was no significant difference in the levels of M1 or M2 macrophages immediately following three cycles of carboplatin relative to vehicle (Fig. 5B and C). M1 macrophages remained at comparable levels throughout the study in both vehicle- and carboplatin-treated mice. M2 macrophages increased over time in the vehicle-treated groups but remained at comparable levels in the carboplatin-treated mice (Fig. 5C). The ratio of M1 marker expressing macrophages relative to M2 marker expressing macrophages significantly decreased over time in the vehicle-treated groups as a result of M2 expansion. However, the ratio of M1 to M2 macrophages was equal in the carboplatin-treated group in residual tumors and remained unchanged in regrown tumors (Fig. 5D). These findings suggest that chemotherapy limits the expansion of M2 macrophages over time, which may be due to their enhanced sensitivity to carboplatin relative to M1 (Fig. 3B). We used this model to quantitate single M1 and M2 surface markers as we wanted to assess the feasibility of detecting differences in a simplified subcutaneous model that provides easily resectable tumors for analysis (27). This model, however, does not take into account the role of tissue resident macrophages which have been linked to the spread of ovarian cancer and the promotion of CSC phenotypes (28).

**Figure 6:**
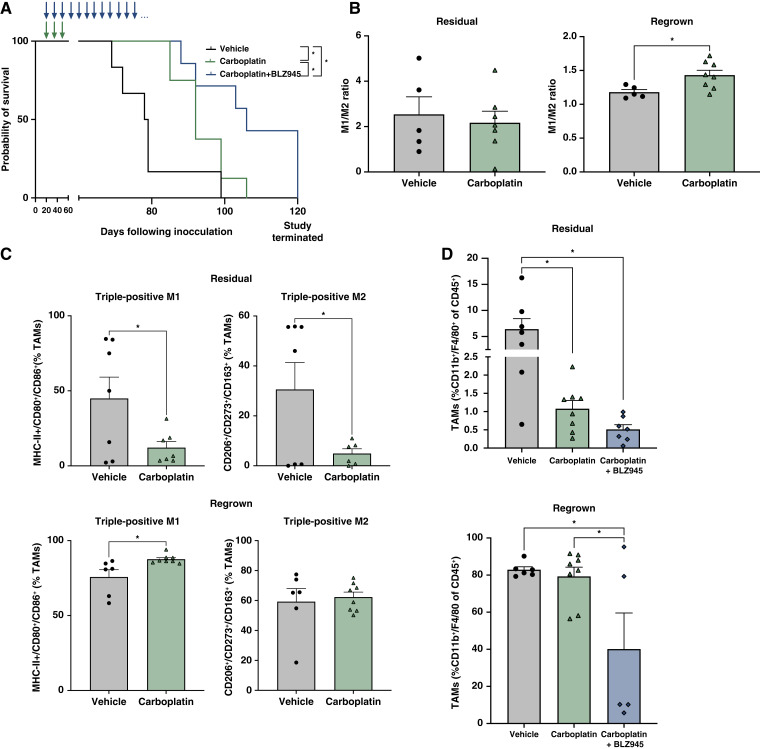
M1/M2 ratio increases in regrown tumors following chemotherapy administration. Mice were inoculated with CAOV4 cells and treated with either vehicle, carboplatin, or carboplatin plus BLZ945. Mice were sacrificed 3 days following four cycles of carboplatin (residual) or allowed to reach endpoint criteria (regrown). Tumors were disassociated for analysis of TAM populations (CD45^+^CD11b^+^F4/80^+^), M1-like (MHC-II^+^CD80^+^CD86^+^) and M2-like (CD163^+^CD206^+^CD273^+^) phenotypes. **A,** Kaplan–Meier survival analysis of mice treated with either vehicle, carboplatin, or carboplatin plus BLZ945 treatment. **B,** Residual (left) and regrown (right) M1/M2 ratio calculated using percent triple positive, as determined by flow cytometry. Unpaired *t* test. **C,** Residual (top) and regrown (bottom) flow cytometry analysis of *in vivo* tumors measuring triple-positive for M1-like phenotypes (MHC-II^+^, CD80^+^, and CD86^+^) or M2-like phenotypes (CD206^+^, CD273^+^, and CD163^+^). Unpaired *t* test. **D,** TAM (CD45^+^CD11b^+^F4/80^+^) changes with *in vivo* chemotherapy with or without TAM inhibition. One-way ANOVA and Tukey *post hoc* test. Mean and SD. *, *P* < 0.05, no stars indicate ns.

We expanded these studies in an intraperitoneal xenograft mouse model of ovarian cancer which has been shown to appropriately recapitulate both resident and infiltrating macrophages in metastatic cancer (27). We inhibited macrophage recruitment with administration of a CSF-R1 inhibitor, BLZ945. Briefly, mice were intraperitoneal injected with CAOV4 cancer cells and 4 days later received weekly intraperitoneal administration of either vehicle, carboplatin, or carboplatin plus BLZ945 by gavage, for 4 weeks. As expected, mice receiving carboplatin had improved survival relative to vehicle. Interestingly inclusion of BLZ945 significantly improved survival over carboplatin alone (Fig. 6A) suggesting that macrophages contribute to disease progression in the presence of carboplatin. Three days following the last carboplatin treatment, a cohort of mice was sacrificed, and peritoneal tissues were resected and analyzed to assess changes in CSCs and two subsequent panels of TAM populations (CD45^+^CD11b^+^F4/80^+^): M1-like (MHC-II^+^, CD80^+^, CD86^+^) and M2-like (CD206^+^, CD273^+^, CD163^+^) populations. Similar to the subcutaneous model, we found that the M1/M2 ratio in residual tumors was comparable between the two treatment groups; however, in contrast to the subcutaneous model, the M1/M2 ratio increased in regrown tumors (Fig. 6B). Carboplatin treatment alone led to a loss of both M1-like triple-positive (MHC-II^+^/CD80^+^/CD86^+^) and M2-like triple-positive (CD206^+^/CD273^+^/CD163^+^) phenotypes (Fig. 6C; for individual markers see Supplementary Fig. S5). We confirmed that the loss of TAMs with carboplatin was further reduced with BLZ945 treatment in the residual tumor group (Fig. 6D). In the regrown tumor group (which represents tumors collected at least 40 to 60 days following the last carboplatin treatment), the TAM population in carboplatin-treated mice was restored to the same level as vehicle-treated mice, whereas mice with continued BLZ945 administration maintained significantly reduced TAMs (Fig. 6D). These results indicate that carboplatin decreases both M1-like and M2-like TAM populations, and suppression of TAMs during chemotherapy administration improves probability of survival.

**Figure 7:**
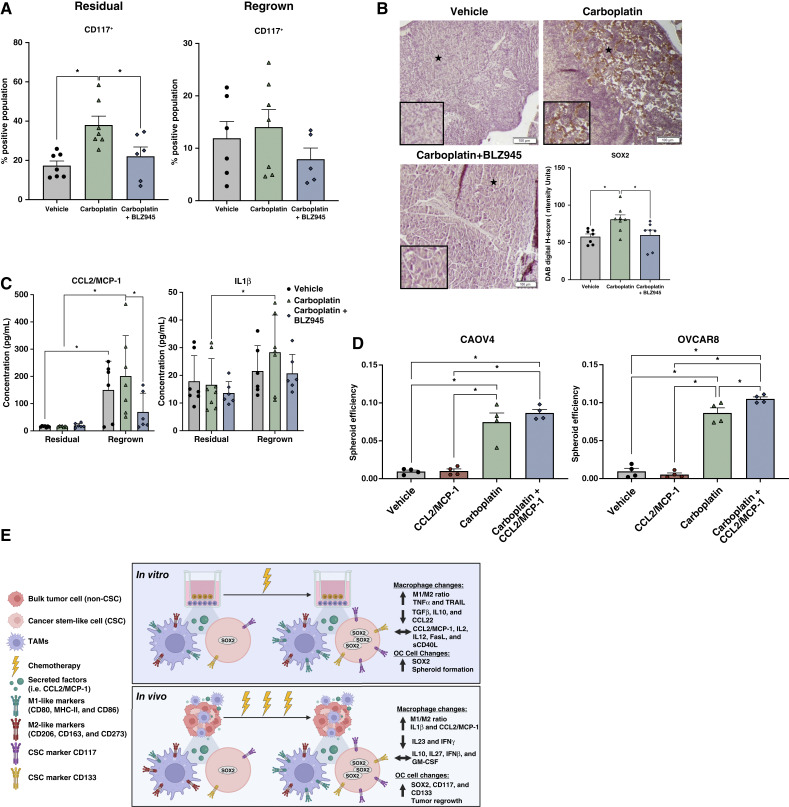
Macrophages promote SOX2 and CD117 expression during chemotherapy administration. Mice were inoculated with CAOV4 cells and treated with either vehicle, carboplatin, or carboplatin plus BLZ945. Mice were sacrificed 3 days following four cycles of carboplatin (residual) or allowed to reach endpoint criteria (regrown). Tumors were dissociated for analysis of CSCs (CD177, CD133, and ALDH). **A,** CSCs (CD117^+^) changes with *in vivo* chemotherapy +/− TAM inhibition. Mean and SD, One-way ANOVA and Tukey *post hoc* test. **B,** Representative images with zoomed inset of regions of interest of (*) in fixed tumor fractions stained for SOX2 (top) and quantification of digital *H*-score using ImageJ (bottom) of residual tumors. One-way ANOVA and Tukey *post hoc* test. **C,** Protein detection via LEGENDplex Mouse Immune Response panel. Two-way ANOVA and Tukey *post hoc* test. **D,** Spheroid formation efficiency in CAOV4 (left) and OVCAR8 (right) cells after 48 hours of treatment with CCL2/MCP-1 (10 ng/mL), carboplatin (275 µmol/L), or in combination. One-way ANOVA and Tukey *post hoc* test. **E,** Consistent changes in expression of surface markers, secreted factors, and cancer cell features following chemotherapy from tested across all populations of macrophages and cancer cells *in vitro* and *in vivo*. Mean and SEM. *, *P* < 0.05, no stars indicate ns. (Created with BioRender.)

To understand whether macrophages are influencing CSCs and SOX2 expression, as our *in vitro* models would suggest, we profiled CD117^+^, CD133^+^, and ALDH^+^ ovarian cancer cells and changes in SOX2 protein expression in the resected tumors from the different treatment groups (Fig. 7A; Supplementary Fig. S6A and S6B). CD117^+^ cells increased with carboplatin treatment in the residual tumor group but became less enriched over time with the regrown tumor group (Fig. 7A). This was expected, as the more proliferative non-CSCs that make up the bulk of the tumor begin to outnumber the more quiescent CSCs. Interestingly, the percentage of CD133^+^ cells remained high in the regrown tumors, suggesting CD133^+^ cells may represent a more proliferative CSC population (Supplementary Fig. S6A and S6B). In both cases however, BLZ945 treatment resulted in a lower percentage of CSCs in both residual tumor and regrown tumor timepoints (Fig. 7A; Supplementary Fig. S6A and S6B). There was no significant effect on ALDH^+^ cells (Supplementary Fig. S6B). We also note that carboplatin treatment led to an increase in SOX2 protein expression in the residual tumors, and this expression was significantly decreased when BLZ945 was also given (Fig. 7B). These findings support the notion that carboplatin enriches for ovarian CSCs, and this is at least partially mediated by TAMs.

We additionally used a mouse-specific cytokine panel for multiplex analysis of changes in immune response cytokines that occurred in the different treatment groups. Interestingly, CCL2/MCP-1 and IL1β were enriched in the carboplatin-treated regrown tumors, and this enrichment was suppressed when BLZ945 was also administered (Fig. 7C). Although CCL2/MCP-1 induces recruitment of monocytes, which may support CSCs in currently unidentified ways, IL1β is a known inducer of stemness in other cancers and may also be important in ovarian cancer. Given these data, we assessed if CCL2/MCP-1 influenced ovarian cancer cell stemness pathways and spheroid formation (Fig. 7D; Supplementary Fig. S6C and S6D). We found that ovarian cancer cells treated with carboplatin in combination with CCL2/MCP-1 for 48 hours had increased *SOX2*, *OCT4*, and *NANOG* gene expression (Supplementary Fig. S6C and S6D) and greater spheroid formation ability relative to carboplatin or CCL2/MCP-1 treatment alone (Fig. 7D). Although there were relatively few changes in the concentrations of cytokines in the residual tumor group, several cytokines were increased in tumors collected from regrown tumors, including IL23, IL6, IL12p70, and IFNγ; however, these were not necessarily changed with BLZ945 relative to carboplatin alone (Supplementary Fig. S7). Taken together, these findings suggest that although carboplatin leads to a modulated secretome and an overall reduction in TAMs, it leads to an enrichment of M1-like TAMs relative to M2-like TAMs that may support CSCs and disease progression (Fig. 7E).

## Discussion

In this study, we sought to characterize changes in macrophage phenotypes in response to carboplatin to elucidate potential mechanisms by which TAMs support CSC maintenance. TAMs comprise almost half of ovarian tumors, and previous studies suggest that they support ovarian CSCs (29). However, the mechanisms of TAM-mediated CSC maintenance are unclear, especially in the context of chemotherapy, in which TAMs are responsive to changes in the tumor. Moreover, this is a relevant time point for monocyte infiltration into tumors in which carboplatin may directly or indirectly influence their phenotypic responses.

We previously showed that *SOX2*, relative to *OCT4* or *NANOG*, is a better marker of drug-resistant CSCs in ovarian cancer (26). In our current study, we found that THP1-and PBMC-derived macrophages did not promote expression of *SOX2* in ovarian cancer cells when co-cultured for 48 hours without cytotoxic drugs. However, in the presence of carboplatin, there was a significant increase in *SOX2* expression relative to carboplatin-treated cancer cells cultured alone. As we previously found, *OCT4* and *NANOG* expression followed a similar trend to *SOX2* but were not consistently increased across all cell lines. It has been shown that co-culture of THP1-derived macrophages with ovarian CSCs could promote M2-TAM polarization, and this increased stemness of the cancer cells through IL8 mediated STAT3 signaling (30). In breast cancer, an increase in IL6 secretion from TAMs promoted expression of *SOX2*, *OCT4*, and *NANOG* (31, 32). Similarly, coculturing lung cancer cells with THP1-derived macrophages resulted in significant increases in *SOX2*, *OCT4*, and *NANOG* expression in cancer cells and M2-like polarization of TAMs (33). These studies, however, were completed in the absence of chemotherapy, and it is unclear whether this would reflect early- or late-stage disease in which the number of M2-like macrophages is limited or expansive, respectively. Moreover, it is likely that whereas both M1-like and M2-like macrophages support CSCs features, the CSCs may induce the expansion of M2-like TAMs important for disease progression. Importantly, these findings indicate that macrophages support stem cell transcription factor expression in several models, and our findings suggest that this is relevant during chemotherapy exposure and involves a proinflammatory response from macrophages.

Recent co-culture studies show that cisplatin or carboplatin can promote M2 polarization through the secretion of proinflammatory IL6 from ovarian cancer cells (34, 35), whereas in other studies, cisplatin was found to promote migration of ovarian cancer cells via M1-like macrophage activity (36). Although there was no change in M1 marker expression in both THP1 and PBMCs, there was an increase in the production of CCL20 and IL1β that subsequently enhanced ovarian cancer cell migration (36). The discrepancies in M1- versus M2-driven cancer cell features may be explained by the inherent plasticity of macrophages and the heterogeneity of TAMs, which express both M1 and M2 markers. Activated TAMs were previously described as being M2-like based on a Th-1 or Th-2 inflammatory response (37, 38); however, more recent studies demonstrate that TAMs share M1 and M2 signatures, both of which can serve protumorigenic functions supporting tumor growth, metastasis, and immune suppression (16, 21). In our intraperitoneal mouse model, we found that TAMs express a variety of M1 and M2 markers, which is corroborated in other studies showing macrophages exist in a continuum of various functional states, leading to a variety of phenotypic responses (38). Although much of this plasticity has been studied in co-culture models with cancer cells, there is limited evaluation of this plasticity in response to chemotherapeutic drugs, which may have a more significant influence on recurrence.

Our findings that M1-like macrophages were more resistant to apoptosis in response to carboplatin and that the M1/M2 ratio increased are in agreement with previous studies showing that M2-like macrophages are more susceptible to platinum drugs relative to M1-like macrophages (34). In response to carboplatin, we found that THP1-derived macrophages increased expression of the M1 surface marker CD80 on M0 or M2-like macrophages, whereas PBMC-derived macrophage decreased expression of the M2 surface marker CD206 on M0, M1-like, and M2-like macrophages. Moreover, both THP1-derived and PBMC-derived macrophages increased M1-like gene expression and secreted factors in response to carboplatin treatment, including TNFα and IL1β, whereas PBMC-derived macrophages simultaneously decreased M2-like secreted factors in response to carboplatin treatment, including IL10 and TGFβ1. A similar study in breast cancer showed that PBMC-derived macrophages treated with cancer cell conditioned media and cisplatin had increased IFNγ, IL6, and TNFα signaling, pathways typically associated with M1-like phenotypes (39). This supports the notion that chemotherapy has the potential to re-educate macrophages toward a proinflammatory phenotype, as our findings also suggest. These data corroborate previous work in patient samples showing reduced expression of markers associated with M2 macrophages and increased expression of proinflammatory pathways and inflammasome activation following paclitaxel and carboplatin treatment (40). Clinical data show that an enrichment of M1-like TAMs and a higher M1/M2 ratio correlates with better overall and progression-free survival in high-grade serous ovarian cancer (41, 42), and this ratio decreases with advanced stage. Our study provides novel insight into phenotypic changes endured by M0, M1-like, or M2-like macrophages immediately following chemotherapy and proposes that proinflammatory cytokines, although effective at eliminating bulk tumor cells, may activate stemness pathways in drug-resistant CSCs to enhance their survival and maintenance during chemotherapy exposure.

In this study, we investigated changes in TAMs, their secreted factors, and corresponding CSC markers using an *in vivo* human xenograft model of ovarian cancer. A total of nine different TAM markers and a panel of secreted factors were evaluated to better assess the complexity of TAM subtypes now identified by single-cell sequencing experiments (24, 25). Analysis of regrown tumors showed that chemotherapy increased M1/M2 ratio and some proinflammatory secreted factors, including CCL2/MCP-1 and IL1β, while decreasing other proinflammatory secreted factors, including IFNγ and IL12p70. It remains unknown how long the high M1/M2 ratio persists clinically relevant scenarios. Nevertheless, our data suggest TAM plasticity in response to chemotherapy, possibly indicating a switch from more interferon enriched TAMs to more inflammatory enriched TAMs as suggested by other studies (25). Although chemotherapy alone significantly depleted the TAM population, it enriched for CD117^+^ and/or CD133^+^ expressing ovarian cancer cells, with a similar trend in ALDH^+^ cells, and this effect was lost when TAMs were depleted. Moreover, depletion of TAMs during chemotherapy prolonged survival relative to chemotherapy alone, suggesting a role for infiltrating TAMs in disease progression during treatment. We also found that chemotherapy-induced increase in proinflammatory cytokines CCL2/MCP-1 and IL1β is lost when TAMs are depleted. Studies in breast cancer suggest blockade of CSF-1 limits macrophage infiltration and improves response to chemotherapy (43–45), and another group (46) found that targeting TAMs by CSF-1R inhibition in a transgenic breast cancer mice model stimulated an intratumoral type I IFN response, enhancing the efficacy of platinum-based chemotherapy. It has also been reported that CCL2/MCP-1 is increased after paclitaxel–carboplatin combination treatment, resulting in macrophage recruitment into ovarian tumors and inhibition of CCL2/MCP-1 increases response to chemotherapy (47, 48). Our data suggest that this may be due to an enrichment of CSCs during chemotherapy, perhaps mediated by CCL2/MCP-1. Ovarian cancer cells treated *in vitro* with CCL2/MCP-1 and carboplatin significantly increased spheroid formation ability and increased *SOX2* expression, suggesting that CCL2/MCP-1 could be one of the secreted factors from macrophages during chemotherapy that supports CSC features. The impact of chemotherapy-induced infiltration of monocytes into the tumor is not fully understood, but it likely facilitates removal of dying tumor cells, which is beneficial; however, the secretory activities involved may enrich for CSCs, which in the long run may facilitate recurrence. As discussed above, several proinflammatory cytokines have been shown to enrich for CSCs that support disease progression following chemotherapy (32, 49, 50). A better understanding of the effects of different TAM populations on CSCs relative to bulk tumor cells may help clarify ovarian cancer biology and disease progression.

The use of immunodeficient mice is both a strength and a limitation of our study. We sought to understand changes in macrophage phenotypes induced directly by chemotherapy, and the immunodeficient model most closely resembled the design of our *in vitro* studies. This model excluded other features of the TME, such as adaptive immune cells; however, macrophages remain the predominant immune cell type in ovarian tumors (51–53), especially during late-stage disease when chemotherapy is essential. Another consideration is the role of tissue-resident versus tumor-infiltrating macrophages, which are not differentiated in this study. Recent studies have highlighted the complex interactions between these populations and how they contribute to CSC features and ovarian cancer progression; nonetheless, marker distinction between tissue-resident and tumor-infiltrating macrophages remains challenging (28, 54). Future work could investigate the impact of each of these macrophage populations and their role in CSC promotion. Our findings provide insight into chemotherapy-mediated changes in M1-like TAMs which, to the best of our knowledge, has not been investigated in relapse models studying ovarian CSCs. In summary, our work demonstrates a role for TAMs in supporting CSCs and disease progression, and our findings suggest that this is due to an increase in M1 polarization in response to carboplatin. Further research into the immediate and long-term effects of cytotoxic therapies on TAM phenotypes will improve our understanding of CSC maintenance and identify novel targets for preventing ovarian cancer recurrence.

## Supplementary Material

Supplemental Figure 1 Macrophage polarization analysis

Supplemental Figure 2 complementary co-culture experiments

Supplemental Figure 3 Baseline secretory profile

Supplemental Figure 4 Carboplatin-induced secretory changes

Supplemental Figure 5 Flow cytometry analysis of ip TAMs

Supplemental Figure 6 Analysis of ip CSCs and CCL2/MCP-1 treatment

Supplemental Figure 7 Mouse Immune Panel
