# Supplementary material for: Chemotherapy Enriches for Proinflammatory Macrophage Phenotypes that Support Cancer Stem-Like Cells and Disease Progression in Ovarian Cancer
Source: Cancer Res Commun. 2024 Oct 9;4(10):2638–52. doi: 10.1158/2767-9764.CRC-24-0311 (PMC11464072; doi:10.1158/2767-9764.CRC-24-0311)
Supplement: Supplemental Figure 1 — Macrophage polarization analysis [file crc-24-0311_supplemental_figure_1_suppsf1.pptx]

## Slide 1
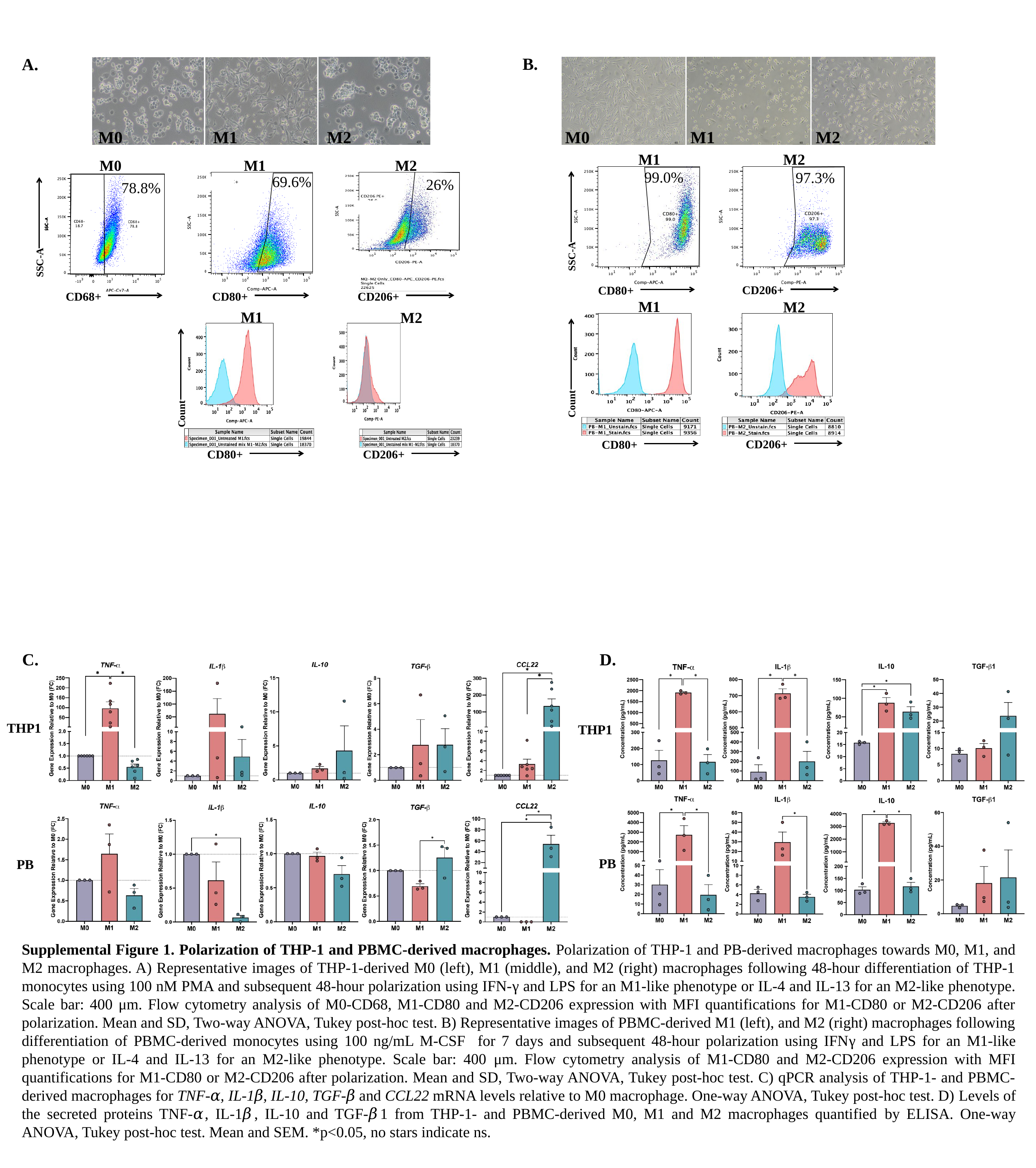

B.
A.
M2
M1
M0
M0
M1
M2
M1
99.0%
CD80+
M2
97.3%
CD206+
SSC-A
M0
M1
69.6%
CD80+
M2
26%
CD206+
78.8%
SSC-A
CD68+
M1
CD80+
M2
CD206+
Count
M1
M2
Count
CD206+
CD80+
C.
D.
THP1
THP1
PB
PB
Supplemental Figure 1. Polarization of THP-1 and PBMC-derived macrophages. Polarization of THP-1 and PB-derived macrophages towards M0, M1, and M2 macrophages. A) Representative images of THP-1-derived M0 (left), M1 (middle), and M2 (right) macrophages following 48-hour differentiation of THP-1 monocytes using 100 nM PMA and subsequent 48-hour polarization using IFN-γ and LPS for an M1-like phenotype or IL-4 and IL-13 for an M2-like phenotype. Scale bar: 400 μm. Flow cytometry analysis of M0-CD68, M1-CD80 and M2-CD206 expression with MFI quantifications for M1-CD80 or M2-CD206 after polarization. Mean and SD, Two-way ANOVA, Tukey post-hoc test. B) Representative images of PBMC-derived M1 (left), and M2 (right) macrophages following differentiation of PBMC-derived monocytes using 100 ng/mL M-CSF for 7 days and subsequent 48-hour polarization using IFNγ and LPS for an M1-like phenotype or IL-4 and IL-13 for an M2-like phenotype. Scale bar: 400 μm. Flow cytometry analysis of M1-CD80 and M2-CD206 expression with MFI quantifications for M1-CD80 or M2-CD206 after polarization. Mean and SD, Two-way ANOVA, Tukey post-hoc test. C) qPCR analysis of THP-1- and PBMC-derived macrophages for TNF-𝛼, IL-1𝛽, IL-10, TGF-𝛽 and CCL22 mRNA levels relative to M0 macrophage. One-way ANOVA, Tukey post-hoc test. D) Levels of the secreted proteins TNF-𝛼, IL-1𝛽, IL-10 and TGF-𝛽1 from THP-1- and PBMC-derived M0, M1 and M2 macrophages quantified by ELISA. One-way ANOVA, Tukey post-hoc test. Mean and SEM. *p<0.05, no stars indicate ns.
