# Supplementary material for: Chemotherapy Enriches for Proinflammatory Macrophage Phenotypes that Support Cancer Stem-Like Cells and Disease Progression in Ovarian Cancer
Source: Cancer Res Commun. 2024 Oct 9;4(10):2638–52. doi: 10.1158/2767-9764.CRC-24-0311 (PMC11464072; doi:10.1158/2767-9764.CRC-24-0311)
Supplement: Supplemental Figure 2 — complementary co-culture experiments [file crc-24-0311_supplemental_figure_2_suppsf2.pptx]

## Slide 1
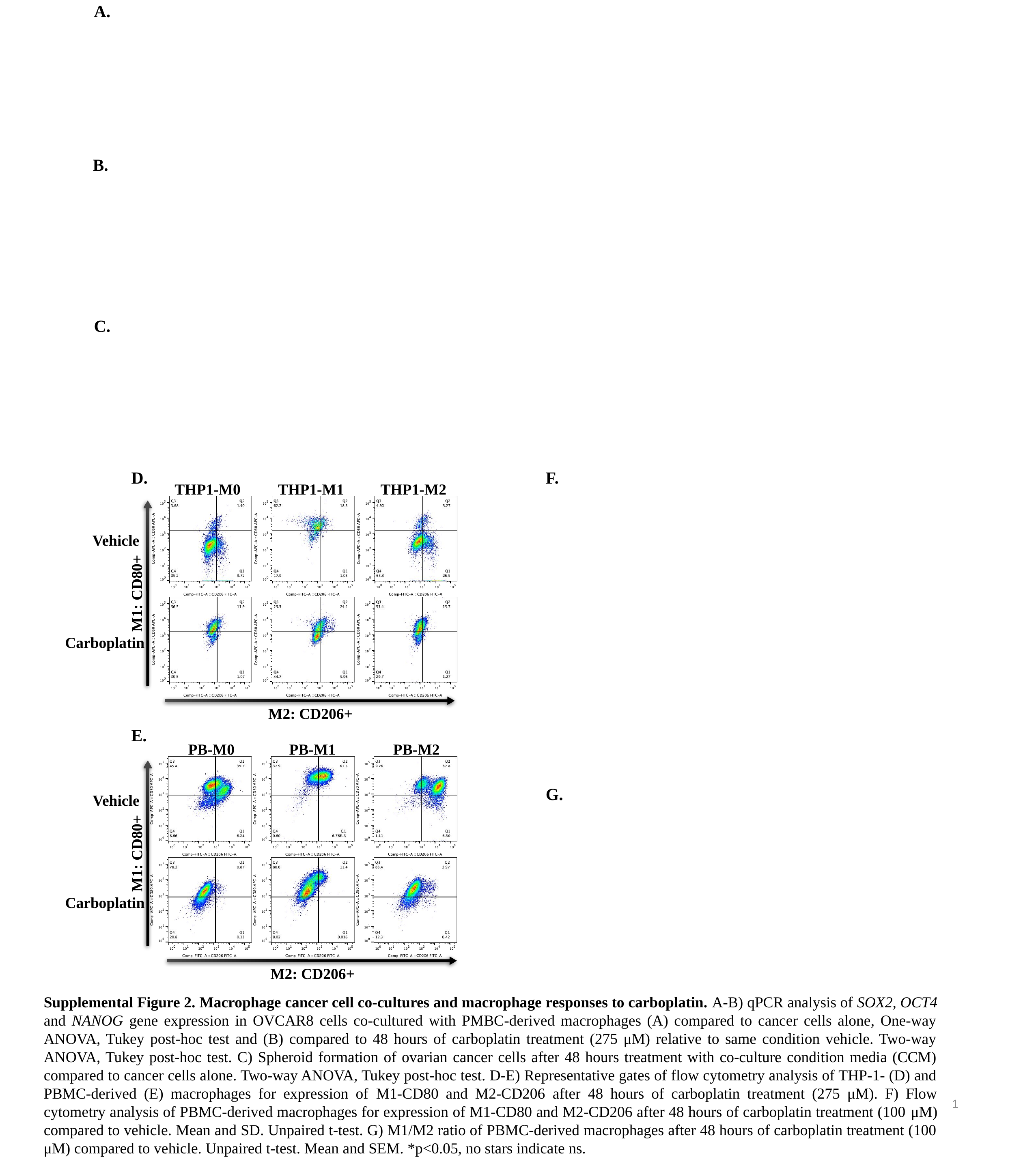

A.
B.
C.
D.
F.
THP1-M0
THP1-M1
THP1-M2
M1: CD80+
Vehicle
Carboplatin
M2: CD206+
E.
PB-M0
PB-M1
PB-M2
M1: CD80+
Vehicle
Carboplatin
M2: CD206+
G.
Supplemental Figure 2. Macrophage cancer cell co-cultures and macrophage responses to carboplatin. A-B) qPCR analysis of SOX2, OCT4 and NANOG gene expression in OVCAR8 cells co-cultured with PMBC-derived macrophages (A) compared to cancer cells alone, One-way ANOVA, Tukey post-hoc test and (B) compared to 48 hours of carboplatin treatment (275 μM) relative to same condition vehicle. Two-way ANOVA, Tukey post-hoc test. C) Spheroid formation of ovarian cancer cells after 48 hours treatment with co-culture condition media (CCM) compared to cancer cells alone. Two-way ANOVA, Tukey post-hoc test. D-E) Representative gates of flow cytometry analysis of THP-1- (D) and PBMC-derived (E) macrophages for expression of M1-CD80 and M2-CD206 after 48 hours of carboplatin treatment (275 μM). F) Flow cytometry analysis of PBMC-derived macrophages for expression of M1-CD80 and M2-CD206 after 48 hours of carboplatin treatment (100 μM) compared to vehicle. Mean and SD. Unpaired t-test. G) M1/M2 ratio of PBMC-derived macrophages after 48 hours of carboplatin treatment (100 μM) compared to vehicle. Unpaired t-test. Mean and SEM. *p<0.05, no stars indicate ns.
1
