# Supplementary material for: Chemotherapy Enriches for Proinflammatory Macrophage Phenotypes that Support Cancer Stem-Like Cells and Disease Progression in Ovarian Cancer
Source: Cancer Res Commun. 2024 Oct 9;4(10):2638–52. doi: 10.1158/2767-9764.CRC-24-0311 (PMC11464072; doi:10.1158/2767-9764.CRC-24-0311)
Supplement: Supplemental Figure 3 — Baseline secretory profile [file crc-24-0311_supplemental_figure_3_suppsf3.pptx]

## Slide 1
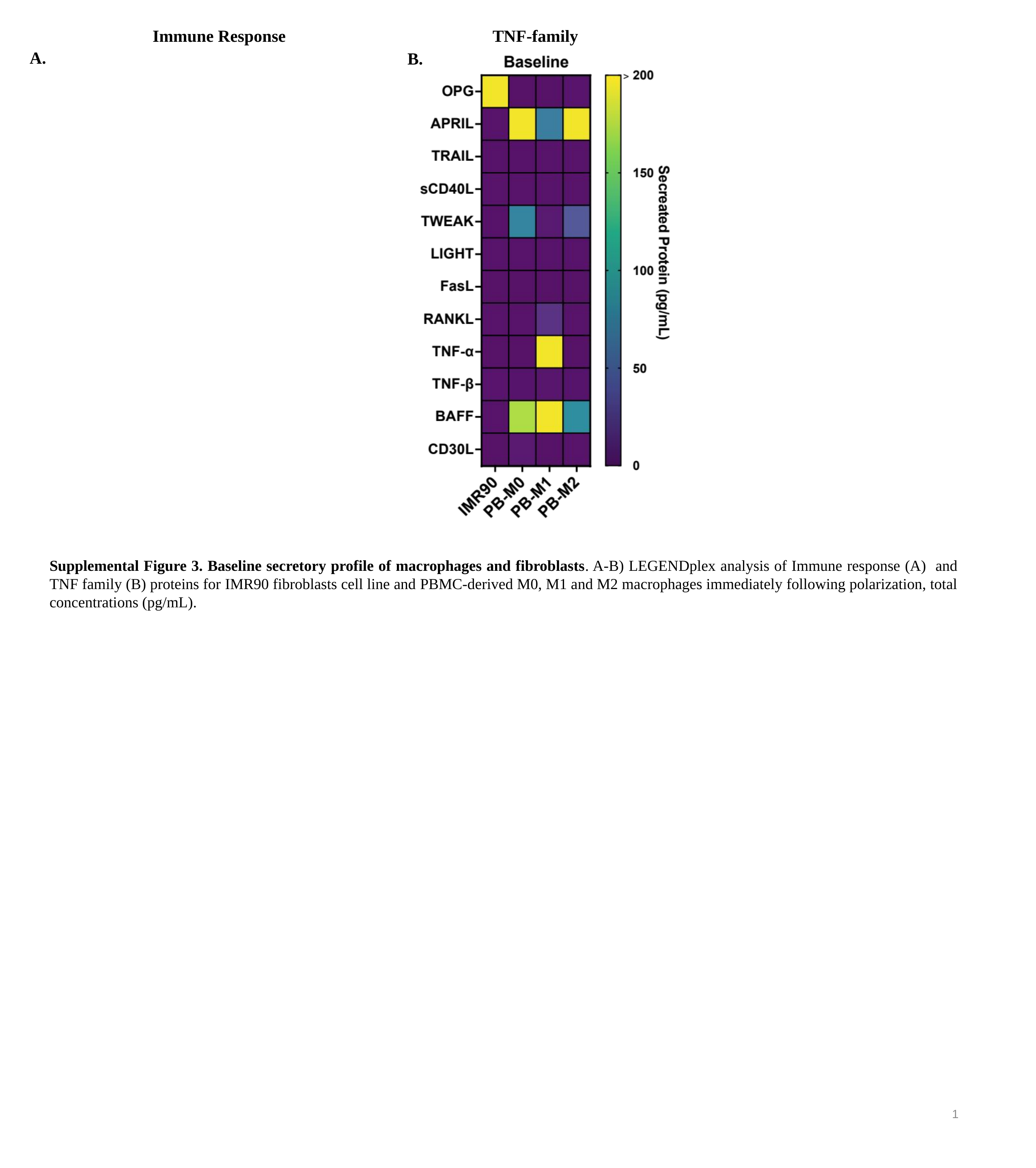

Immune Response
TNF-family
A.
B.
Supplemental Figure 3. Baseline secretory profile of macrophages and fibroblasts. A-B) LEGENDplex analysis of Immune response (A) and TNF family (B) proteins for IMR90 fibroblasts cell line and PBMC-derived M0, M1 and M2 macrophages immediately following polarization, total concentrations (pg/mL).
1
