# Supplementary material for: Chemotherapy Enriches for Proinflammatory Macrophage Phenotypes that Support Cancer Stem-Like Cells and Disease Progression in Ovarian Cancer
Source: Cancer Res Commun. 2024 Oct 9;4(10):2638–52. doi: 10.1158/2767-9764.CRC-24-0311 (PMC11464072; doi:10.1158/2767-9764.CRC-24-0311)
Supplement: Supplemental Figure 4 — Carboplatin-induced secretory changes [file crc-24-0311_supplemental_figure_4_suppsf4.pptx]

## Slide 1
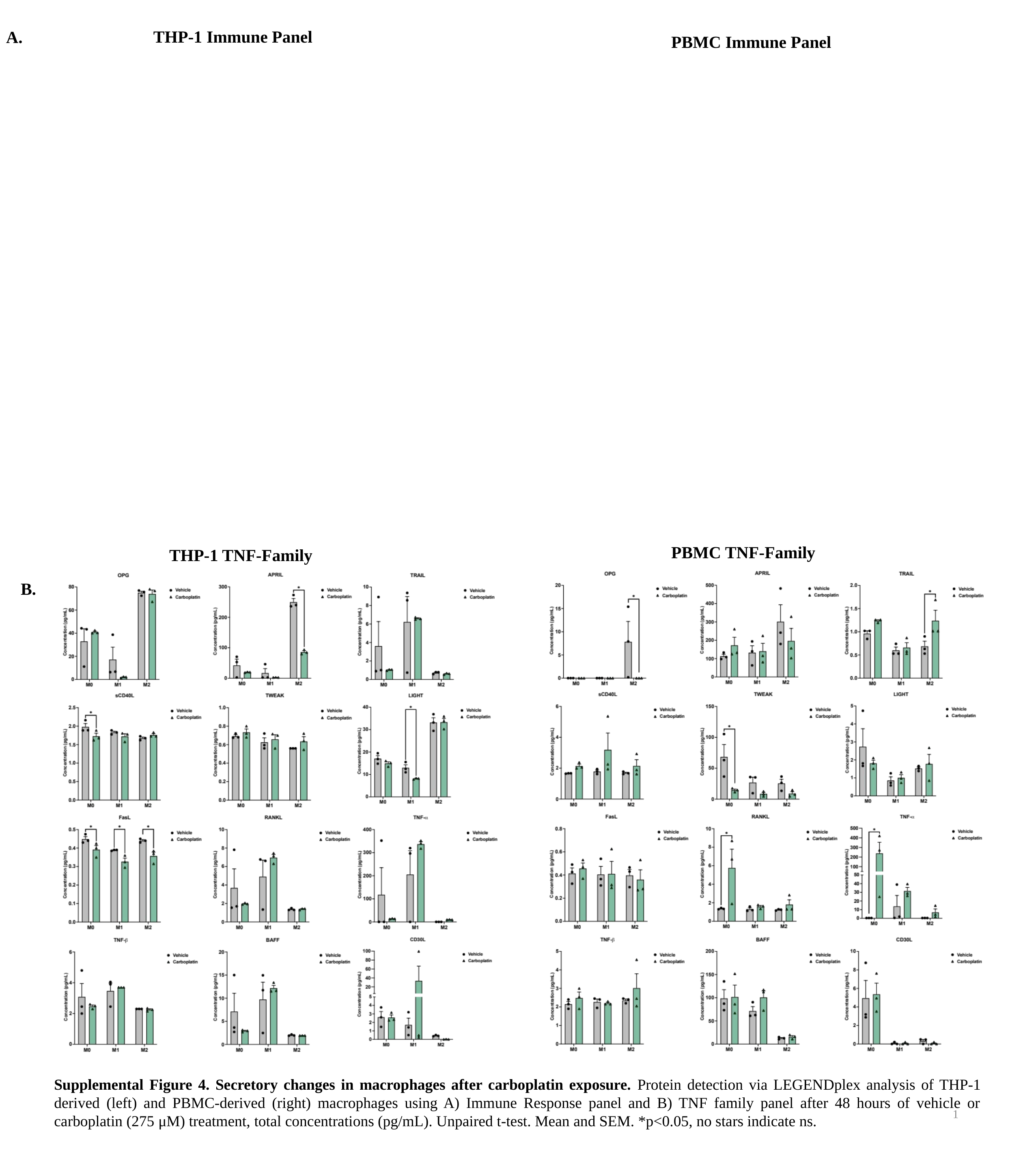

THP-1 Immune Panel
A.
PBMC Immune Panel
PBMC TNF-Family
THP-1 TNF-Family
B.
Supplemental Figure 4. Secretory changes in macrophages after carboplatin exposure. Protein detection via LEGENDplex analysis of THP-1 derived (left) and PBMC-derived (right) macrophages using A) Immune Response panel and B) TNF family panel after 48 hours of vehicle or carboplatin (275 μM) treatment, total concentrations (pg/mL). Unpaired t-test. Mean and SEM. *p<0.05, no stars indicate ns.
1
