# Supplementary material for: Chemotherapy Enriches for Proinflammatory Macrophage Phenotypes that Support Cancer Stem-Like Cells and Disease Progression in Ovarian Cancer
Source: Cancer Res Commun. 2024 Oct 9;4(10):2638–52. doi: 10.1158/2767-9764.CRC-24-0311 (PMC11464072; doi:10.1158/2767-9764.CRC-24-0311)
Supplement: Supplemental Figure 5 — Flow cytometry analysis of ip TAMs [file crc-24-0311_supplemental_figure_5_suppsf5.pptx]

## Slide 1
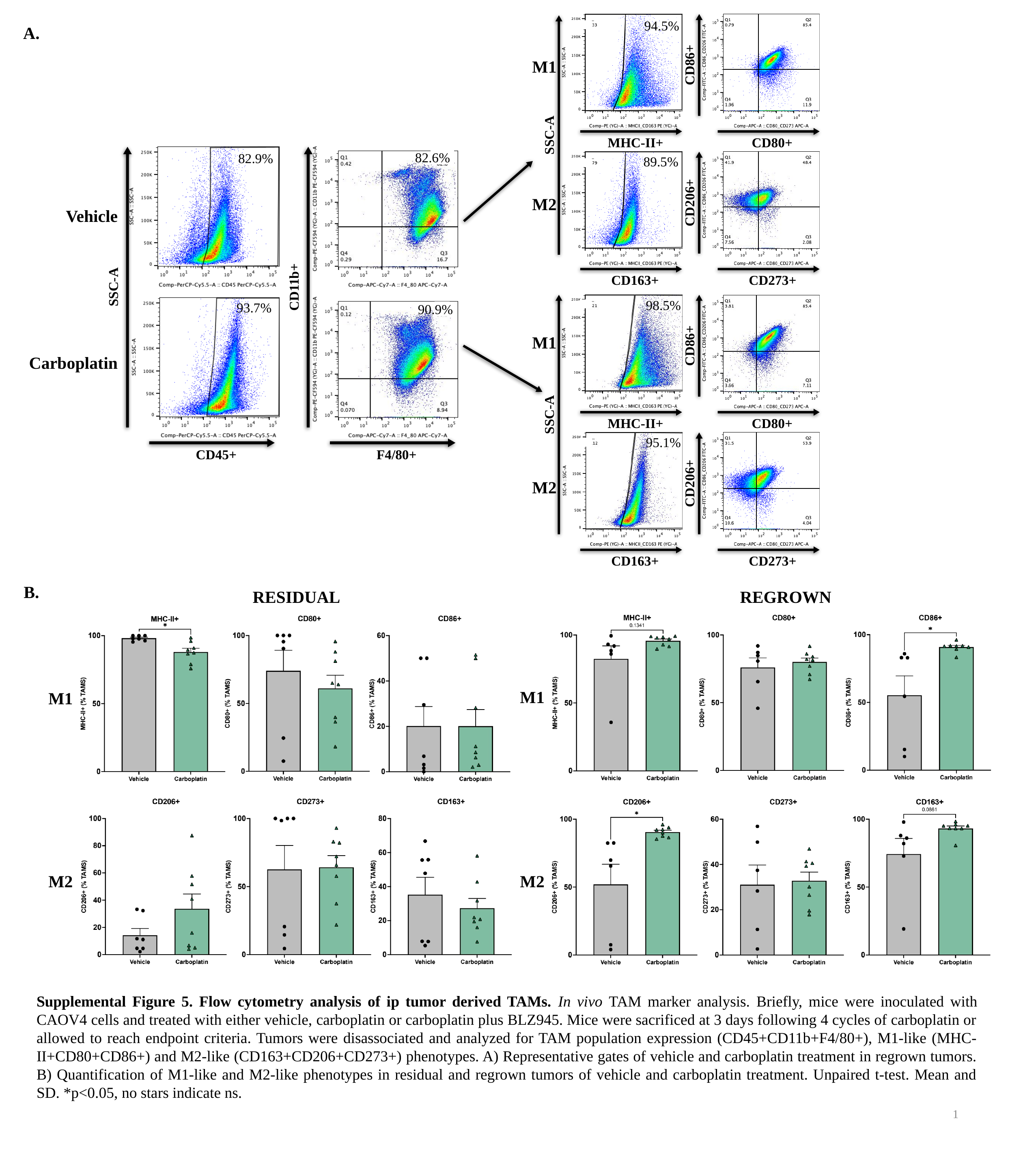

94.5%
CD86+
CD80+
SSC-A
A.
M1
MHC-II+
82.6%
SSC-A
82.9%
CD11b+
Vehicle
93.7%
90.9%
Carboplatin
CD45+
F4/80+
89.5%
CD206+
CD273+
M2
CD163+
98.5%
SSC-A
CD86+
CD80+
M1
MHC-II+
95.1%
CD206+
CD273+
M2
CD163+
B.
RESIDUAL
M1
M2
REGROWN
M1
M2
Supplemental Figure 5. Flow cytometry analysis of ip tumor derived TAMs. In vivo TAM marker analysis. Briefly, mice were inoculated with CAOV4 cells and treated with either vehicle, carboplatin or carboplatin plus BLZ945. Mice were sacrificed at 3 days following 4 cycles of carboplatin or allowed to reach endpoint criteria. Tumors were disassociated and analyzed for TAM population expression (CD45+CD11b+F4/80+), M1-like (MHC-II+CD80+CD86+) and M2-like (CD163+CD206+CD273+) phenotypes. A) Representative gates of vehicle and carboplatin treatment in regrown tumors. B) Quantification of M1-like and M2-like phenotypes in residual and regrown tumors of vehicle and carboplatin treatment. Unpaired t-test. Mean and SD. *p<0.05, no stars indicate ns.
1
