# Supplementary material for: Chemotherapy Enriches for Proinflammatory Macrophage Phenotypes that Support Cancer Stem-Like Cells and Disease Progression in Ovarian Cancer
Source: Cancer Res Commun. 2024 Oct 9;4(10):2638–52. doi: 10.1158/2767-9764.CRC-24-0311 (PMC11464072; doi:10.1158/2767-9764.CRC-24-0311)
Supplement: Supplemental Figure 6 — Analysis of ip CSCs and CCL2/MCP-1 treatment [file crc-24-0311_supplemental_figure_6_suppsf6.pptx]

## Slide 1
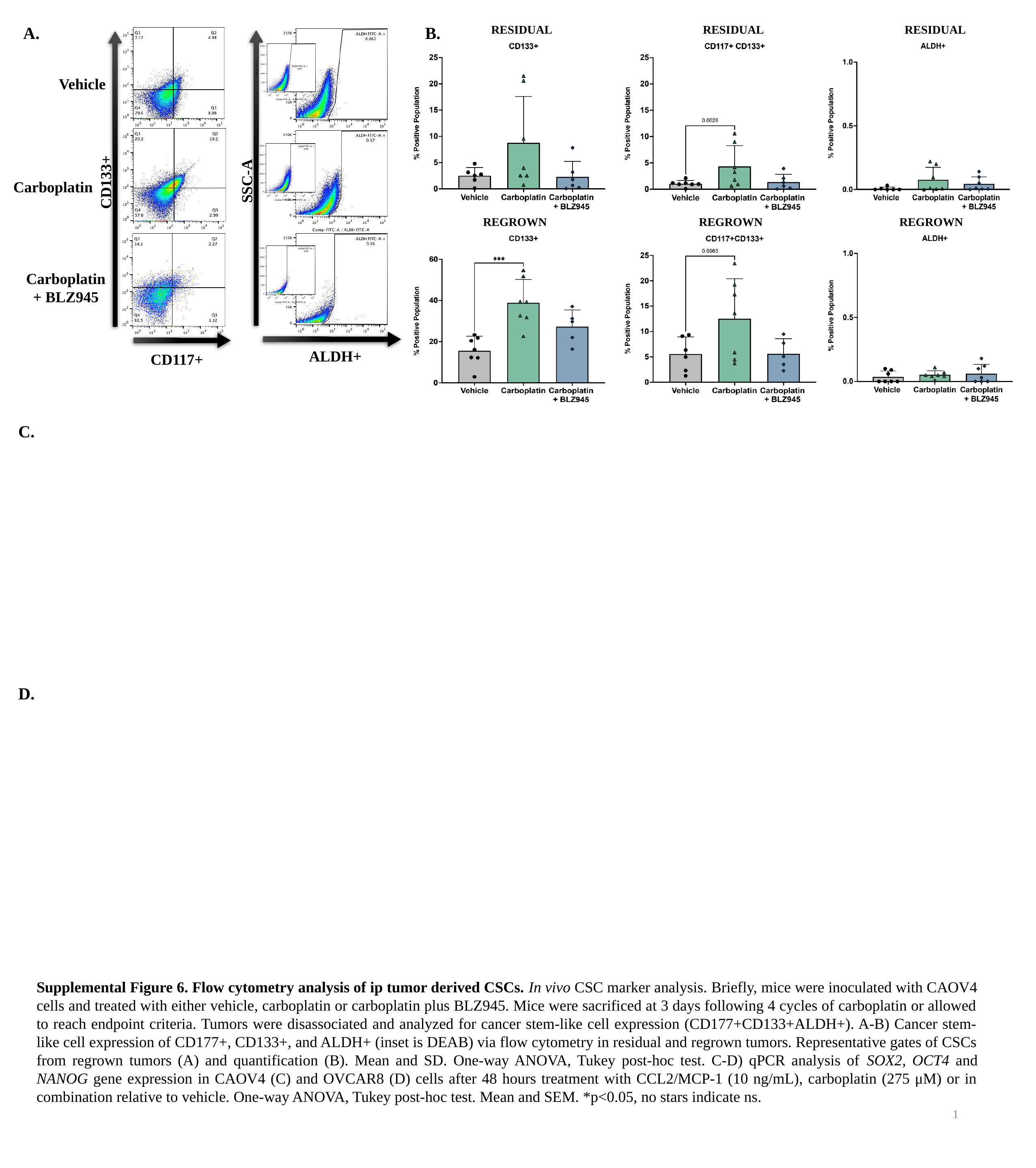

A.
RESIDUAL
RESIDUAL
RESIDUAL
REGROWN
REGROWN
REGROWN
B.
SSC-A
CD133+
Vehicle
Carboplatin
Carboplatin
+ BLZ945
ALDH+
CD117+
C.
D.
Supplemental Figure 6. Flow cytometry analysis of ip tumor derived CSCs. In vivo CSC marker analysis. Briefly, mice were inoculated with CAOV4 cells and treated with either vehicle, carboplatin or carboplatin plus BLZ945. Mice were sacrificed at 3 days following 4 cycles of carboplatin or allowed to reach endpoint criteria. Tumors were disassociated and analyzed for cancer stem-like cell expression (CD177+CD133+ALDH+). A-B) Cancer stem-like cell expression of CD177+, CD133+, and ALDH+ (inset is DEAB) via flow cytometry in residual and regrown tumors. Representative gates of CSCs from regrown tumors (A) and quantification (B). Mean and SD. One-way ANOVA, Tukey post-hoc test. C-D) qPCR analysis of SOX2, OCT4 and NANOG gene expression in CAOV4 (C) and OVCAR8 (D) cells after 48 hours treatment with CCL2/MCP-1 (10 ng/mL), carboplatin (275 μM) or in combination relative to vehicle. One-way ANOVA, Tukey post-hoc test. Mean and SEM. *p<0.05, no stars indicate ns.
1
