# Supplementary material for: Chemotherapy Enriches for Proinflammatory Macrophage Phenotypes that Support Cancer Stem-Like Cells and Disease Progression in Ovarian Cancer
Source: Cancer Res Commun. 2024 Oct 9;4(10):2638–52. doi: 10.1158/2767-9764.CRC-24-0311 (PMC11464072; doi:10.1158/2767-9764.CRC-24-0311)
Supplement: Supplemental Figure 7 — Mouse Immune Panel [file crc-24-0311_supplemental_figure_7_suppsf7.pptx]

## Slide 1
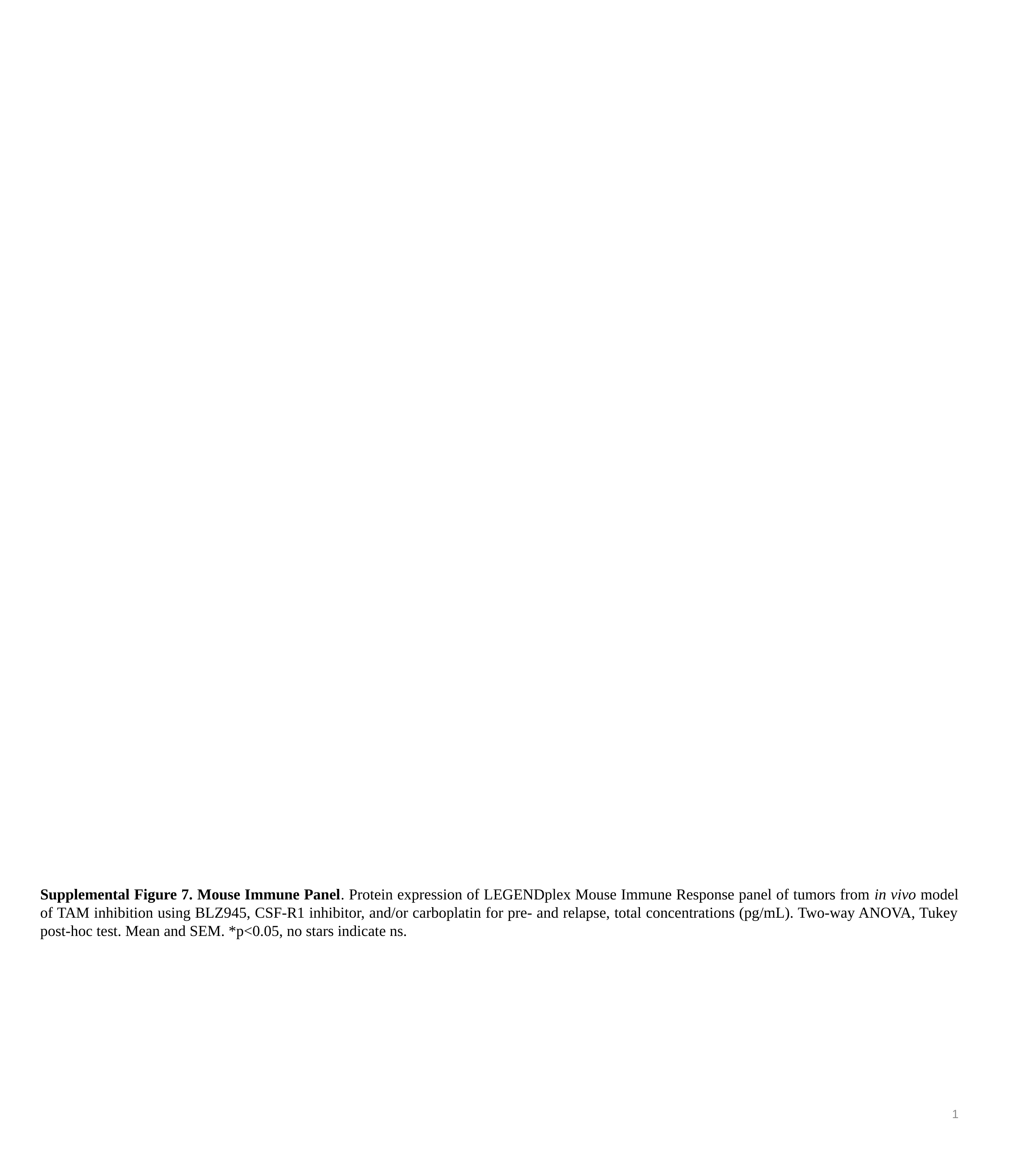

Supplemental Figure 7. Mouse Immune Panel. Protein expression of LEGENDplex Mouse Immune Response panel of tumors from in vivo model of TAM inhibition using BLZ945, CSF-R1 inhibitor, and/or carboplatin for pre- and relapse, total concentrations (pg/mL). Two-way ANOVA, Tukey post-hoc test. Mean and SEM. *p<0.05, no stars indicate ns.
1
